# Comparison of Phytochemical Contents, Antioxidant and Antibacterial Activities of Various Solvent Extracts Obtained from ‘Maluma’ Avocado Pulp Powder

**DOI:** 10.3390/molecules26247693

**Published:** 2021-12-20

**Authors:** Thi-Van-Linh Nguyen, Quoc-Duy Nguyen, Nhu-Ngoc Nguyen, Thi-Thuy-Dung Nguyen

**Affiliations:** Faculty of Environmental and Food Engineering, Nguyen Tat Thanh University, Ho Chi Minh City 754000, Vietnam; nqduy@ntt.edu.vn (Q.-D.N.); nnngoc@ntt.edu.vn (N.-N.N.); dungnnt@ntt.edu.vn (T.-T.-D.N.)

**Keywords:** antioxidant activity, antibacterial activity, avocado powder, total carotenoids, total chlorophylls, total polyphenols

## Abstract

Although avocado is a superfood rich in phytochemicals with high antioxidant activities, studies on the antibacterial properties of its pulp are limited, except for seed and peel portions. In this study, three types of solvent (acetone, methanol, and diethyl ether) were used to obtain the extracts from “Maluma” avocado pulp powder prepared by infrared drying. The extracts were analyzed for total polyphenols, phytopigments (total chlorophylls and carotenoids), antioxidant activities (ferric-reducing antioxidant power (FRAP), 2,2-Diphenyl-1-picrylhydrazyl (DPPH), and 2,2-azino-bis(3-ethylbenzothiazoline-6-sulfonic acid (ABTS) assays), and antibacterial activities against seven pathogens (*Shigella sonnei* ATCC 9290, *Escherichia coli* ATCC 8739, *Salmonella typhi* ATCC 6539, *Vibrio parahaemolyticus* ATCC 17802, *Proteus mirabilis* ATCC 25933, *Staphylococcus aureus* ATCC 6538, and *Bacillus cereus* ATCC 11778). The results showed that the acetone solvent could extract the highest polyphenols and chlorophylls with the highest antioxidant activity in terms of ABTS and DPPH assays. In contrast, diethyl ether exhibited the most significant content of carotenoids and FRAP values. However, the methanol extract was the best solvent, exerting the strongest antibacterial and meaningful antioxidant activities. For the bacterial activities, Gram-positive pathogens (*Bacillus cereus* and *Staphylococcus aureus*) were inhibited more efficiently by avocado extracts than Gram-negative bacteria. Therefore, the extracts from avocado powder showed great potential for applications in food processing and preservation, pharmaceuticals, and cosmetics.

## 1. Introduction

Plants are a rich source of phytochemical compounds such as flavonoids, alkaloids, tannins, and terpenoids, which have antibacterial and antioxidant properties [1]. Antioxidant activity from natural compounds has been shown to protect the human body from the effects of free radicals, preventing oxidative stress and related diseases [2,3,4]. Meanwhile, plant extracts with antibacterial ability are promising and safe sources for applications as additives for food production [5,6,7] or aquaculture [8,9,10]. Therefore, many studies have focused on extracting and evaluating antibacterial activities of herb, vegetable, and fruit extracts, such as clove [11], peppermint [12], perilla (*Perilla frutescens* L.) [13], fish mint [14], pineapple [15], and various seed extracts [16]. It is shown that plant extracts have broad-spectrum antibacterial activity, being effective against both Gram-positive and Gram-negative bacteria, fungi, and parasites [17,18]. While cinnamon leaves, garlic, basil leaves, curry leaves, ginger root, and mustard exhibit antibacterial properties against a wide range of both Gram-negative and Gram-positive bacteria (*Bacillus cereus*, *Staphylococcus aureus*, *Listeria monocytogenes*, *Escherichia coli* (*E. coli*), and *Salmonella infantis*) [19], extracts of cruciferous vegetables show resistance against Gram-negative bacteria [20]. Besides, the application of innovative techniques such as biosynthesis of nanoparticles using extracts with antioxidant and antibaterial activity could enhance antibacterial properties [21,22]. 

Avocado is a prospective fruit that has received minimal attention among plant materials of interest for antioxidant and antibacterial activities, while this fruit has a high concentration of phenolics, ascorbic acid, carotenoids, vitamin E, and flavonoids, which are known for antioxidation actions [23]. However, compared to avocado flesh, its waste has been commonly utilized in various types of research on antioxidant capacity and antibacterial activity; for example, avocado seeds have been proven to be a significant source of phenolic compounds with antioxidant characteristics. Avocado seeds have a high concentration of antibacterial compounds, which has sparked interest in the investigation of extracting antibacterial components from avocado seeds [24]. Meanwhile, the avocado’s lipid fraction is high in phytochemicals and antioxidants [25]. Egbuonu et al. also demonstrated the antibacterial activity of avocado seed extracts as illustrated by the diameter of the inhibition zone against *Proteus mirabilis* (23 ± 0.14 mm), *Staphylococcus aureus* (16 ± 0.04 mm), and *Pseudomonas aeruginosa* (15 ± 0.11 mm), despite being lower than that of ciprofloxacin antibiotics [26]. Besides, this extract also shows higher antifungal activity against *Aspergillus niger* than the ketoconazole [24], with higher total phenolic content and antioxidant capacity [27]. 

Little research has been conducted on avocado flesh, especially on its antibacterial properties. Rodríguez-Carpena et al. analyzed the antioxidant activity and antibacterial potential of ethyl acetate (70%), acetone (70%), and methanol (70%) extracts of avocado pulp, peel, and seed. They concluded that the peels and seeds of two avocado varieties, namely, “Hass” and “Fuerte” contain higher amounts of phenolics and antioxidant activity than the avocado pulp, and they have moderate activity against Gram-positive bacteria [28]. In a recent study, the powdered “Maluma” avocado pulp with the highest retention of bioactive compounds and antioxidant activities was successfully produced using the infrared technique [29]. Infrared drying was considered an innovative technique that brings more benefits in the implementation and high retention of dried product quality [30,31]. After eliminating the moisture, the materials could enhance the stability of quality during storage, be more uniform as well as improving the efficiency of extraction. To the best of our knowledge, the biological activity of “Maluma” avocado has not yet published. Therefore, the purpose of this study was to investigate the effects of different avocado extracts obtained from avocado powder using acetone, methanol, and diethyl ether as solvents on polyphenols, chlorophylls, carotenoids, antioxidant activity, and antibacterial activity.

## 2. Results

**Figure 1:**
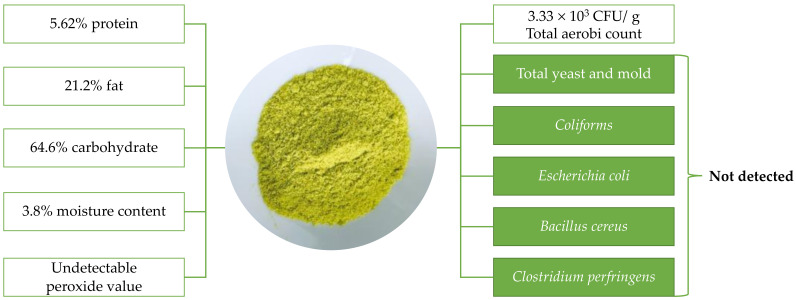
Physicochemical and microbiological properties of avocado powder prepared from avocado flesh (*Persea Americana* Mill. cv. “Maluma”).

The physicochemical and microbiological properties of the avocado powder are shown in Figure 1. 

**Figure 2:**
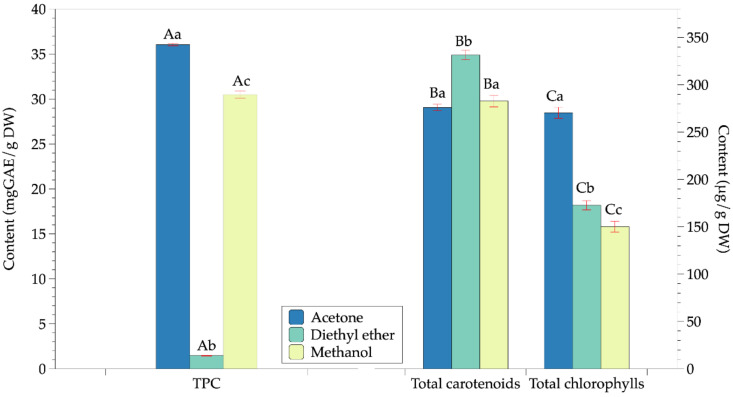
Effects of solvents on the total phenolics content (TPC) and the content of phytopigments of avocado extracts. Note: capital letters represent the same group of responding variables, and values within a group with the same lowercase letters are not significantly different (*p* > 0.05).

**Figure 3:**
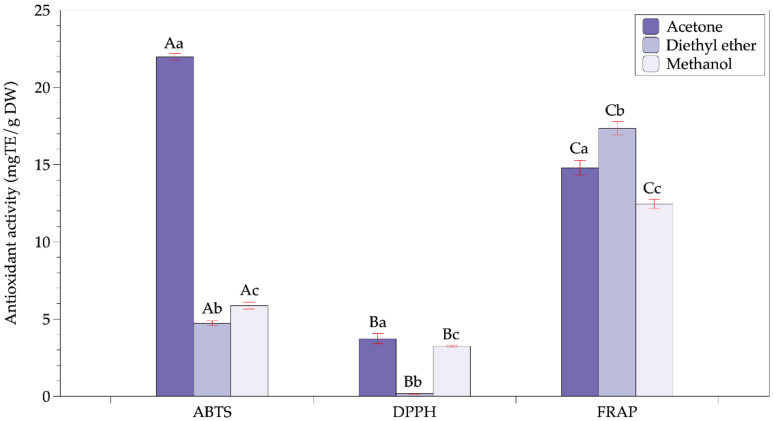
Effects of solvents on the antioxidant activity of avocado extracts. Note: capital letters represent the same group of responding variables, and values within a group with the same lowercase letters are not significantly different (*p* > 0.05).

Avocado powder prepared by infrared drying obtained good quality with undetectable peroxide value and the group of pathogenic microorganisms. In addition, the total aerobic count (3.3 × 10^3^ colony-forming units (CFU)/g) was smaller than the microbiological standard of the dried foods (10^6^ CFU/g) [31]. Besides, the moisture content of avocado powder was lowered to below 4% that could assure the stability of the product during storage [32]. Then, this powder was extracted with a different type of solvent, including acetone, methanol, and diethyl ether. The effects of other solvents on the content of phytopigments and the total phenolics content (TPC) are shown in Figure 2. In addition, the antioxidant activities of avocado extracts corresponding to different solvents are presented in Figure 3.

The results showed that the TPC and the total chlorophylls content of the avocado extracts with acetone had the most significant value. However, the diethyl ether extracts obtained the highest value of total carotenoids. The methanolic extract also showed a high content of TPC and phytopigments (total chlorophylls and carotenoids). Specifically, the extract with methanol had a higher TPC than diethyl ether extract. In addition, the methanolic extract obtained a similar content of carotenoids as the acetone extract. 

Although the methanol extract had a TPC value, ferric-reducing antioxidant power (FRAP), and 2,2-Diphenyl-1-picrylhydrazyl (DPPH) activity comparable to that of the acetone extract, it showed a significantly low radical scavenging capacity in terms of 2,2-azino-bis(3-ethylbenzothiazoline-6-sulfonic acid (ABTS). The diethyl ether extract exhibited the highest FRAP iron reduction capacity (17.35 mg Trolox equivalent (TE)/g of sample dry weight (DW)) despite being the lowest TPC. Plus, the extract with diethyl ether also possessed the lowest DPPH radical scavenging capacity. Overall, acetone was shown to be the best solvent to obtain phenolic compounds with high phytopigments and antioxidant activity. 

The correlation between the chemical composition (TPC and phytopigments) and the antioxidant activity of avocado extracts is shown in Table 1**.** The results showed that the carotenoids had a high positive correlation with the ferric-reducing antioxidant power. Meanwhile, the TPC had a robust positive correlation with the DPPH radical scavenging activity and a moderate positive correlation with ABTS activity. Table 1 also shows the total chlorophylls had a very high positive correlation with ABTS cation-scavenging activity. It could be concluded that TPC and phytopigments made significant contributions with different antioxidant mechanisms to the antioxidant capacity of avocados.

**Figure 4:**
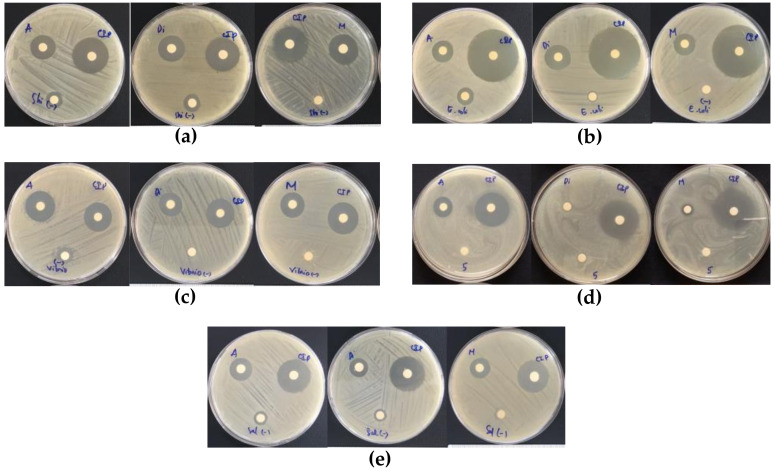
Visual observations of the inhibitory effects of avocado extracts against Gram-negative pathogens including *Shigella sonnei* ATCC 9290 (**a**), *Escherichia coli* ATCC 8739 (**b**), *Vibrio parahaemolyticus* ATCC 17802 (**c**), *Proteus mirabilis* ATCC 25933 (**d**), and *Salmonella typhi* ATCC 6539 (**e**). Note: A, Di, M, CIP, and (–) symbols on petri discs denote the acetone, diethyl ether, methanol extracts, ciprofloxacin (reference antibiotics), and negative control, respectively.

**Figure 5:**
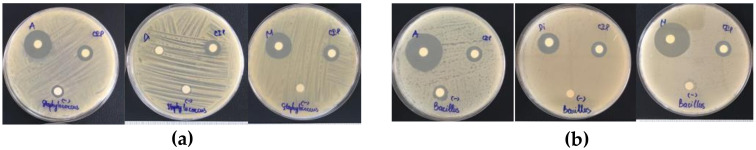
Visual observations of the inhibitory effects of avocado extracts against Gram-positive pathogens including *Staphylococcus aureus* ATCC 6538 (**a**) and *Bacillus cereus* ATCC 11778 (**b**). Note: A, Di, M, CIP, and (–) symbols on petri discs denote the acetone, diethyl ether, methanol extracts, ciprofloxacin (reference antibiotics), and negative control, respectively.

The effects of different solvents on the antibacterial activities of avocado extracts are shown in Figure 4 and Figure 5. The diameters of inhibition zones of avocado extracts using an agar well diffusion assay are presented in Table 2.

The results showed that the methanol and acetone extracts had the ability to inhibit seven bacterial strains. Specifically, the methanol extract had the best antibacterial activity with an average inhibition zone diameter from 10 mm against *Proteus mirabilis* to 28 mm against *Bacillus cereus*, followed by the acetone extract with those values from 5 mm against *Salmonella typhi* to 17 mm against *Bacillus cereus*. The diethyl ether extract was able to inhibit five out of seven tested bacterial strains (except *Proteus mirabilis* and *Staphylococcus aureus*), with the diameter of inhibition zones being in the range of 3–15 mm. It can be concluded that the type of solvent had a significant impact on the antibacterial activity of avocado extracts. Besides, the antibacterial compounds in avocado powder have a broad spectrum against both Gram-positive and Gram-negative bacteria.

## 3. Discussion

### 3.1. Physico-Chemical and Microbiological Properties of Avocado Powder

Avocado flesh is well-known as a material that faces difficulty in removing moisture until it reaches the state of easy to grind to obtain the powder structure. In this study, powder from avocado flesh was prepared by infrared drying, in which the combination of the innovative drying technology as infrared drying and the avocado pulp with a thin layer (2 mm) showed effectiveness in removing moisture from materials, as illustrated by the low moisture content of the resulting avocado powder (3.8% on the wet basis). During the drying, the evaporation process happened rapidly, leading to the exceptional appearance of avocado powder (see Figure 1). Additionally, the drying process consumed a short time (50 min) to complete, causing the hydrolysis of lipids hardly to occur to release fatty acids, the reactants in the initiation phase of lipid peroxidation. As a consequence, the peroxide value was undetected in avocado powder. 

### 3.2. Effect of Solvents on the Phytochemical Contents of Avocado Extracts

Acetone, methanol, and diethyl ether are among the common solvents used to extract active ingredients from plants with moderate to high polarity [33,34]. Methanol is more effective in extracting polyphenols with higher extraction yields of phenolics, flavonoids, and tannins than acetone and diethyl ether [35,36]. Moreover, acetone is suitable for the extraction of flavanols [37,38]. In this study, the TPC was significantly changed under the variation of solvent types, because the polarity of the solvent would have a significant impact on the solubility of soluble [39,40] through the differences in the different dielectric constants of the solvents (20.7, 32.7, and 4.3 for acetone, methanol, and diethyl ether). The solubility of polyphenols in the solvent is mainly dependent on the molecular size and structure of TPC, as well as the presence and distribution of hydroxyl groups [41]. Moreover, the hydrogen bonds between polyphenols and proteins that exist in the plant structure would also impact TPC extraction. Thus, the solvent with higher polarity would extract more TPC. It is the main reason why aqueous solvents such as methanol and acetone extract TPC efficiently. Some previous studies confirmed that acetone is the best solvent for extracting polyphenols in other plants [40,42,43]. However, still existed the opposite results such as in the work of Iloki-Assanga et al. [44] or Dailey and Vuong [45]. Iloki-Assanga et al. observed that acetone shows the best solvent for the extraction of leaves *Bucida buceras* and trunk *B. buceras*, but methanolic extraction obtained the highest total phenols content in extracting from Stem *B. buceras*, Oak *Phoradendron californicum*, and Mesquite *P. californicum*, [44]. Dailey and Vuong showed that methanol extracts more TPC and antioxidant activity from skin waste than acetone [45]. 

It was reported that organic solvents as acetone, chloroform, hexane, isopropanol, methanol, methylene chloride, and diethyl ether could extract carotenoids. Carotenoids and chlorophylls were identified to belong to lipophilic pigments, in which the total carotenoids including both polar and nonpolar carotenoids exist [46,47]. Meanwhile, chlorophyll was considered amphipathic molecules [46]. Therefore, diethyl ether extracts the highest of both total carotenoids and chlorophylls. Despite the lower contents of total carotenoids and chlorophylls in acetone and methanol extracts, these extracts are comparable to diethyl ether extract. 

### 3.3. Effect of Solvents on the Antioxidant Activities of Avocado Extracts

In the plants, TPC and phytopigments (such as carotenoids and chlorophylls) are proved to make the major contribution to antioxidant activity. Polyphenols and phytopigments (such as chlorophylls and carotenoids) could act as antioxidants. Wang et al. also concluded that phenolic compounds in the pulp account for 13% of the total phenolic content, contributing to only 5% of the antioxidant capacity of the whole avocado, and pigments such as chlorophylls and carotenoids make a minor contribution to the antioxidant capacity of avocados [27].

The TPC is the critical component in the evaluation of the antioxidant activity of plant extracts. The redox properties of TPC are characterized, such as the ability to reduce agents, hydrogen donors, singlet oxygen quenchers, and metal chelators [48,49,50]. This study also found the TPC has strong and moderate correlations with DPPH and ABTS activity, respectively. Thus, the TPC shows the high ability to scavenge DPPH radicals and the formation of an oxidized radical cation. The numerous studies on plant extracts also similarly observed the positive correlation between the TPC and antioxidant activity with DPPH and ABTS activity [51,52,53]. However, some opposite findings reported no correlation between the TPC and antioxidant activity in extracts of some food and medicinal plants [54,55]. The reason could be that the content, structure, and interaction between bioactive compounds significantly impact antioxidant activity [56]. 

Carotenoids are well-known as antioxidants due to the redox properties and the structure of carotenoids. Some mechanisms are determined to characterize the antioxidant activity of carotenoids, such as electron transfer, hydrogen abstraction/reduction, and formation of carotenoid–radical adducts [47]. In the FRAP assay, the antioxidant would reduce the Fe^+3^/TPTZ complex to the ferrous form based on the electron donors of antioxidant compounds [44]. That clearly explains why the total carotenoids have a high positive correlation with FRAP activity (r = 0.77974; *p* < 0.05). 

Chlorophylls could perform the antioxidant activity with two mechanisms such as anti-radical capacity and donations of the electron [57,58,59] based on the ideal structure and configuration. In this study, the findings showed an extremely high correlation with ABTS activity (r = 0.9595; *p* < 0.05), but no correlation with FRAP activity (r = 0.14305; *p* > 0.05). 

Finally, the results indicated that none of the tested bioactive compounds could represent antioxidant activity in extracts. However, it could conclude that the TPC and phytopigments (such as carotenoids and chlorophylls) made significant contributions to the antioxidant activity of extracts with different antioxidant mechanisms. 

### 3.4. Effect of Solvents on the Antibacterial Activities of Avocado Extracts

In plants, the secondary metabolites known as phytochemical compounds show biological activities [60]. Among these activities, the antibacterial activities of phytochemical compounds have received great interest in evaluating the biological activities of plant extracts. The antibacterial compounds in the avocado powder were found to have a broad spectrum against both Gram-positive and Gram-negative bacteria (see Figure 4 and Figure 5). In this study, the results showed the existence of carotenoids and the TPC in avocado powder. Carotenoids in plant extracts were found to be effective against *Staphylococcus aureus* ATCC 6538, *Bacillus cereus* 13061, *E. coli* ATCC 25922, and *Salmonella typhimurium* ATCC 13,311 [61]. However, the exact antibacterial mechanisms of carotenoids are still limited. The TPC could be extracted from different parts of plants such as leaves [62], peels [63], seed [64], and kernel [65]. In addition, the TPC shows the ability of inhibition against bacteria such as *Staphylococcus aureus, Enterobacter aerogenes, Salmonella typhi*, *and Klebsiella pneumoniae*, *Bacillus subtilis*, *Pseudomonas aeruginosa*, *Providencia stuartii*, *Propionibacterium acnes*, and *Staphylococcus epidermidis*. The TPC possesses antibacterial activity based on direct effects on the life process of microorganisms. The TPC could inactivate hydrolytic enzymes [66] or interact with enzymes [67]. Moreover, the TPC also attacks the structure of the cell or gives resistance in the metabolic activity of microorganisms through binding protein molecules as cell envelope transport proteins [66], adsorption [62], or disruption to cell membranes [68], etc. Therefore, it clearly explains why extracts with a higher TPC, such as acetone and methanol extracts, have better inhibition of bacterial than diethyl ether extracts with the lowest TPC. In this study, the extract with methanol showed the best inhibition of the tested bacteria compared to acetone and diethyl ether extracts, except *Proteus mirabilis*. The main reason is that the methanol has been shown to be effective in extracting polyphenols, phytopigments, and antioxidant activity. In addition, it may be due to methanol extracts also containing significant other compounds with antibacterial activity. In addition, it can be noted that acetone and diethyl ether also exhibited antibacterial activity against most of the investigated bacteria. 

Some studies have determined that the compositions of antibacterial substances found in plants such as phenolics, fatty acids, and terpenes interact with enzymes and proteins of bacterial cell membranes, leading to the cell death or inhibition of bacterial enzymes for amino acid synthesis [69,70,71]. Therefore, the difference in the structure of the cell walls between Gram-positive bacteria and Gram-negative bacteria, in which Gram-negative bacteria have the presence of an outer membrane, which acts as a barrier, limiting the diffusion of compounds through the bacterial lipopolysaccharide layer [72]. That well explains why Gram-positive bacteria (*Bacillus cereus* and *Staphylococcus aureus*) are more sensitive to the extracts than Gram-negative bacteria.

## 4. Materials and Methods

### 4.1. Material, Chemicals and Microorganisms

#### 4.1.1. Material

Fresh avocado (*Persea Americana* Mill. cv. “Maluma”) was received from a local farm in DakLak, Viet Nam; only fully ripe avocado with a green surface was chosen.

#### 4.1.2. Chemicals

Gallic acid, 2,4,6-tripyridyl-s-triazine (TPTZ), DPPH, ABTS, and 6-hydroxy-2,5,7,8 tetramethylchroman-2-carboxylic acid (Trolox) were purchased from Sigma-Aldrich (Singapore). Folin–Ciocalteu reagent (2 N) was prepared basically from solid sodium tungstate, sodium molybdate, and lithium sulfate. Maltodextrin (Dextrose equivalent (DE) in the range of 16.5–19.5; Sigma-Aldrich) and ciprofloxacin antibiotics (Sigma-Aldrich) were purchased from a local supplier.

Tryptic soy agar (TSA), plate count agar (PCA), Violet Red Bile with Lactose (VRBL) agar, Tryptone Bile Glucuronic (TBX) agar, Mannitol Egg Yolk (MYP) agar, Tryptose Sulfite Cycloserine (TSC) agar, and Dichloran-Glycerol (DG18) agar were purchased from Hi-Media Laboratory (Mumbai, India).

Acetic acid, sodium acetate, chloroform, potassium iodide, sodium thiosulfate pentahydrate, starch, sodium carbonate, methanol, acetone, diethyl ether, potassium persulfate, phosphoric acid, hydrochloric acid, ferric chloride hexahydrate, and other chemicals were of analytical grade.

#### 4.1.3. Microorganisms

Microorganisms used in this study were provided by NTT Hi-Tech Institute (NHTI, Nguyen Tat Thanh University, Ho Chi Minh City, Vietnam), including five Gram-negative pathogens (*Shigella sonnei* ATCC 9290, *E. coli* ATCC 8739, *Salmonella typhi* ATCC 6539, *Vibrio parahaemolyticus* ATCC 17802, and *Proteus mirabilis* ATCC 25933) and two Gram-positive pathogens (*Staphylococcus aureus* ATCC 6538 and *Bacillus cereus* ATCC 11778). The microorganisms were kept frozen at −20 °C in TSA broth containing glycerol (10%, *v*/*v*) at the Microbiology Laboratory of the Department of Food Technology, Faculty of Environmental and Food Engineering (Nguyen Tat Thanh University). 

### 4.2. Preparation of Avocado Powder and Experimental Design

**Figure 6:**
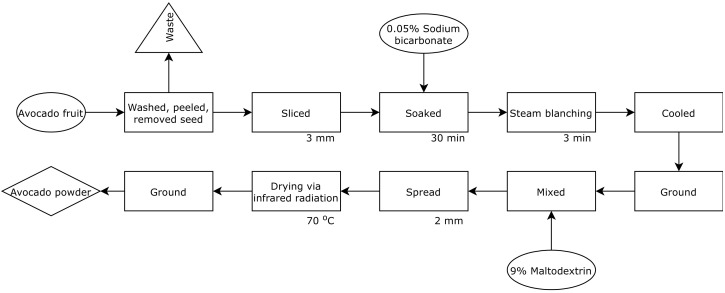
Flowchart showing avocado powder process.

The avocado powder was processed following the steps in Figure 6**.**

The obtained powder was stored at −4 °C until further analysis. The powder was determined the physicochemical properties according to Association of Official Analytical Chemists (AOAC, 2000). The TPC was determined according to the Kjeldahl method (AOAC 991.20). The total fat determination was performed by modified Mojonnier method (AOAC 989.05). The total carbohydrate content was analyzed by difference (AOAC 986.25). The moisture content was determined by the gravimetric method, using a dry air oven until constant weight (AOAC 952.08). The microbiological properties were described by ISO 4833-1:2013 for total aerobic counts (72 h, 30 °C), ISO 4832:2006 for coliform counts (24 h, 37 °C), ISO 16649-2:2001 for *E. coli* counts (24 h, 44 °C), ISO 7932:2004 for *B. cereus* (24 h, 30 °C), ISO 7937:2004 for *Clostridium* *perfringens* (20 h, 37 °C, anaerobic), and ISO 21527-2:2008 for total yeast and mold counts (5–7 days, 25 °C). Microbiological counts were expressed as the number of colony-forming units per gram of sample (CFU/g).

In this study, three types of the absolute solvent, including acetone, methanol, and diethyl ether, were used to investigate chemical properties, antioxidant, and antibacterial activities of the corresponding extracts obtained according to the procedure described in the literature [27]. The sample (0.2 g) was mixed with a 5 mL solvent and then vortexed for 30 s at 2000 rpm using a VELP ZX4 advanced IR vortex mixer (VELP Scientifica, Milan, Italy) to start the extraction step. After sonication (40 KHz, 240 W, 5 min) in a Pro 100 ultrasonic cleaner (Asonic, Ljubljana, Slovenia), the sample was again vortexed for 10 s and cooled for 20 min at 10 °C. The cooled sample was then sonicated for a second time at the previous condition and centrifuged in a PLC-05 centrifuge (Gemmy Industrial Corp., Taipei, Taiwan) at 1220× *g* for 10 min. The supernatant was collected and diluted to 25 mL to analyze the TPC, total carotenoids, total chlorophylls, antioxidant activities (ABTS cation radical scavenging assay, DPPH radical scavenging assay, and FRAP), and antibacterial activity against seven pathogens.

### 4.3. Analysis of Avocado Extracts

#### 4.3.1. TPC

The Folin-Ciocalteu technique, as specified in ISO 14502-1:2005, was used to measure the TPC. Briefly, 0.6 mL of the diluted sample was added to 1.5 mL of Folin-Ciocalteu reagent (10-fold dilution) and incubated in the dark for 10 min to evaluate the TPC. The reaction mixture was incubated in the dark for 60 min, after 1.2 mL of 7.5% (*w*/*v*) Na_2_CO_3_ was added. Using a the UV-9000 spectrometer (Metash, Shanghai, China), the absorbance was measured at 765 nm, with distilled water as a blank. The gallic acid calibration curve was used to determine the TPC, which was reported as mg gallic acid equivalent/g of sample dry weight (mg GAE/g DW).

#### 4.3.2. Phytopigments Content

The solvent in the supernatant was evaporated in a pertri disk using an LO-FS100 forced convection oven (LK Lab, Namyangju, Korea) at 50 °C for 30 min and finally redissolved to 10 mL by pure diethyl ether for the analysis of the total carotenoids and chlorophyll content (μg/g DW), as presented in the literature [73].

#### 4.3.3. ABTS Cation Radical Scavenging Activity

With few changes, the ABTS free radical reduction activity was carried out according to the technique reported in the literature [20]. Frist, 2.85 mL of the stock ABTS reagent was applied to 0.15 mL of the diluted sample to measure the ABTS free radical scavenging activity. Then, the reaction mixture was incubated in the dark for 30 min, and the absorbance was measured at 734 nm using a UV-Vis spectrophotometer against a blank of methanol. The percent inhibition of ABTS free radical inhibition was determined using the following equation: percent inhibition = (1 − absorbance of sample/absorbance of control) × 100. The antioxidant activity of ABTS was determined using the Trolox concentration–% inhibition curve and reported as mg Trolox equivalent/g of sample dry weight (mg TE/g DW).

#### 4.3.4. DPPH Radical Scavenging Activity

The DPPH radical scavenging activity of the extract was determined according to Brand-Williams et al. with minor changes [74]. One-hundred and fifty microliters of the sample was reacted with 2850 μL of DPPH working solution for 30 min under dark conditions. Finally, the sample was measured for absorbance at 515 nm over a methanol blank. DPPH radical scavenging activity was determined using the %DPPH radical inhibition-standard concentration curve (%DPPH radical inhibition = (1 − absorbance of sample/absorbance of blank) × 100) and expressed as mg Trolox equivalent/g of sample dry weight (mg TE/g DW).

#### 4.3.5. FRAP

The FRAP assay was performed according to the method described in the literature with some modifications [75]. First, 2.85 mL of FRAP reagent was added to 0.15 mL of the diluted sample. The reaction mixture was incubated for 30 min in the dark, and the absorbance was measured at 593 nm with a UV-Vis spectrophotometer over distilled water as a blank. The FRAP was calculated based on the Trolox calibration curve and expressed as mg Trolox equivalent/g of sample dry weight (mg TE/g DW).

#### 4.3.6. Antimicrobial Activity by Agar Well Diffusion Assay

Before the test, stock cultures were inoculated into a broth and incubated at 37 °C for 18 h. The antimicrobial activities of the plant extracts were determined by agar well diffusion assay [76]. The bacterial pathogens were grown in a liquid medium for 20 h to yield a final concentration of 10^8^ CFU/mL. Next, aliquots of 0.1 mL of the test microorganisms were spread over the surface of agar plates. Sterile filter paper discs were saturated with 50 μL of plant extracts and ciprofloxacin (0.1 mg/mL) as reference antibiotics. The soaked discs were then placed in the middle of the plates and incubated at 37 °C for 18 h, after which the diameter (unit: mm) of each inhibitory zone was measured as the difference between the diameters of the extracts and the negative control. Negative controls were prepared using the same solvents employed to dissolve the plant extracts.

### 4.4. Statistical Analysis

Using basic statistical techniques, experimental data were analyzed using SPSS 15 software (SPSS Inc., Chicago, IL, USA). One-way ANOVA was used to determine the differences between the samples, and Tukey’s multiple range test was applied to determine the significant differences between mean values at the significance level of 5%. All experiments were conducted in triplicate.

## 5. Conclusions

This study characterized the antioxidant and antibacterial activities of different extracts (acetone, methanol, and diethyl ether) from avocado pulp powder. The results showed these activities were solvent-dependent, in which methanol and acetone were effective in extracting phenolic components and chlorophylls. Still, the extracts with diethyl ether obtained the highest content of carotenoids. The results found that the TPC and phytopigments in the investigated extracts showed different antioxidant mechanisms. Thus, these compounds played an important role in the antioxidant activity of extracts. Except for the diethyl ether extract, the methanol and acetone extracts inhibited all seven pathogens tested. The methanol extract had the most excellent antibacterial activity with exceptional inhibition zones (diameter ranging from 10 to 28 mm). In addition, Gram-positive pathogens (*Bacillus cereus* and *Staphylococcus aureus*) were more sensitive to extracts from avocado powder than Gram-negative pathogens. Finally, it was indicated that the methanol extract was the best solvent, since it possessed the highest antibacterial and significant antioxidant activities. For further study, after the optimal extraction conditions were determined, the extracts with considerable antioxidant and antibacterial activities may have a wide range of applications in various fields, such as food processing and preservation, pharmaceuticals, and cosmetics.

## Figures and Tables

**Table 1:** Pearson correlation coefficients between the phytopigments, TPC, and antioxidant activity of avocado extracts.

	TPC (mg Gallic Acid Equivalent/g of Sample Dry Weight (mg GAE/g DW))	Total Carotenoids (μg/g DW)	Total Chlorophylls (μg/g DW)
2,2-Diphenyl-1-picrylhydrazyl (DPPH; mg Trolox equivalent (TE)/g DW)	**0.99308**	**−0.98586**	0.44616
*p*-value (2-tailed)	0.00000	0.00000	0.22868
2,2-azino-bis(3-ethylbenzothiazoline-6-sulfonic acid (ABTS; mg TE/g DW)	**0.66928**	−0.62861	**0.96595**
*p*-value (2-tailed)	0.04865	0.06979	0.00002
Ferric-reducing antioxidant power (FRAP; mg TE/g DW)	**−0.77974**	**0.78034**	0.14305
*p*-value (2-tailed)	0.01321	0.01309	0.71350

Note: correlations in bold are significant at the 5% level (2-tailed).

**Table 2:** Antibacterial activities of avocado extracts using different solvents against seven pathogens as presented in the diameters of inhibition zones using an agar well diffusion assay.

No.	Pathogen	Sample	Inhibitory Diameter (mm) *		
			Acetone	Diethyl Ether	Methanol
1	*Shigella sonnei* ATCC 9290	Ciprofloxacin	26	26	26
		Extracts	7	7	20
		Negative control	11	11	0
2	*Escherichia coli* ATCC 8739	Ciprofloxacin	32	31	33
		Extracts	6	12	16
		Negative control	11	8	0
3	*Salmonella typhi* ATCC 6539	Ciprofloxacin	25	25	23
		Extracts	5	3	15
		Negative control	12	9	0
4	*Vibrio parahaemolyticus* ATCC 17802	Ciprofloxacin	21	20	21
		Extracts	11	13	18
		Negative control	9	0	0
5	*Proteus mirabilis* ATCC 25933	Ciprofloxacin	26	27	26
		Extracts	14	0	10
		Negative control	0	0	0
6	*Staphylococcus aureus* ATCC 6538	Ciprofloxacin	10	10	10
		Extracts	11	0	20
		Negative control	10	7	0
7	*Bacillus cereus* ATCC 11778	Ciprofloxacin	11	10	11
		Extracts	17	15	28
		Negative control	11	0	0

* The inhibitory diameter of extracts was calculated as the difference between the diameters of the inhibition zones of extracts and negative control. Ciprofloxacin was dissolved in sterile water.

## Data Availability

Data are contained within the article.

## Sample Availability

Not available.
